# Leveraging an Arts-Based Approach to Foster Engagement, Nurture Kindness, and Prevent Violence

**DOI:** 10.3390/bs15060799

**Published:** 2025-06-11

**Authors:** Yok-Fong Paat, Diego Garcia Tovar, Nathan W. Myers, Max C. E. Orezzoli, Anne M. Giangiulio, Sarah L. Ruiz, Angela V. Dorado, Luis R. Torres-Hostos

**Affiliations:** 1 Department of Social Work, The University of Texas at El Paso, El Paso, TX 79968, USA; 2 New Mexico State University, Las Cruces, NM 88003, USA; 3 Department of Social Sciences, Florida Memorial University, Miami Gardens, FL 33054, USA; 4 Department of Art, The University of Texas at El Paso, El Paso, TX 79968, USA; 5 Sun City Dietitians, El Paso, TX 79905, USA; 6 School of Social Work, The University of Texas Rio Grande Valley, Edinburg, TX 78539, USA

**Keywords:** arts-based approaches, violence prevention, community engagement, civic learning, media literacy

## Abstract

Drawing from the insights of community partners, this study explored the roles and benefits of arts-based approaches to foster civic learning, critical media literacy, and community engagement. It also uncovered approaches to promote kindness, prevent violence, and combat online extremism, offering insights into strategies that may enhance community engagement and create a positive impact. We presented our model framework, a detailed case study of our project, and qualitative methods incorporating 15 interviews with our community partners to capture a broad range of perspectives and experiences. Interviewees were community partners who collaborated with our project in organizing events and activities using an arts-based approach to promote kindness, awareness, and violence prevention since the inception of the project. Data were analyzed using thematic data analysis. We categorized the community partners’ responses into four key themes: (1) the inherent benefits of the arts, (2) promoting kindness and preventing violence through artistic expression, (3) teaching civic responsibility through the arts, and (4) practical strategies for collaborating with community partners. The practice implications and lessons learned were discussed.

## 1. Introduction

Incidents of targeted violence, including mass shootings and acts of domestic terrorism, pose a critical threat to public safety and demand urgent attention (Federal Bureau of Investigation, 2021; Center for Prevention Programs and Partnerships, 2024). The Federal Bureau of Investigation (FBI) reported 229 active shooter incidents between 2019 and 2023, which marks an 89% increase compared to the previous five-year period (2014–2018) (Federal Bureau of Investigation, 2024). The impact of targeted violence is profound and encompasses physical harm, psychological trauma, and lasting societal consequences (Soni & Tekin, 2023; Moreland, 2024). Understanding ways to mitigate potential threats may help prevent situations from escalating into dangerous incidents. Addressing the root causes of targeted violence, such as reducing social isolation and preventing radicalization resulting from ideological extremism, is an essential primary prevention strategy in public health. These strategies focus on preventing harmful events and reducing risks before they occur, with the goal of fostering a safer and more resilient community (Bhui et al., 2012; Center for Prevention Programs and Partnerships, 2024). To achieve these, there is a rising need for creative and innovative community-based initiatives that foster collaboration among diverse stakeholders in order to strengthen community connectedness and resilience (Blackburn et al., 2023; U.S. Department of Justice Office of Justice Programs, 2024).

Drawing from the insights of community partners, this study explored the roles and benefits of arts-based approaches to foster civic learning, critical media literacy, and community engagement. It also uncovered approaches to promote kindness, prevent violence, and combat online extremism, offering insights into strategies that may enhance community engagement and create a positive impact. We presented our model framework, a detailed case study of our project, and qualitative methods incorporating 15 interviews with our community partners to capture a broad range of perspectives and experiences. Although a few studies have explored the potential benefits of arts-based interventions in rehabilitation efforts (Edwards et al., 2018; Oren et al., 2019), there is limited evidence on how arts-based approaches can be effectively integrated into violence prevention measures in different community-based projects. While some research has highlighted the positive impact of the arts on mental health and health outcomes (Van Lith et al., 2012; Jensen & Bonde, 2018; Chan et al., 2023), a comprehensive understanding of its integration into prevention and treatment strategies remains insufficient and unclear. Additionally, the voices of community partners and practitioners working with different populations across the lifespan have yet to be adequately investigated in current research.

## 2. Literature Review

### 2.1. Advantages of Implementing an Arts-Based Approach

Engaging in visual and expressive arts provides therapeutic benefits and improves mental health for individuals of all ages (Ho et al., 2020; Morison et al., 2021). In addition to serving as an outlet for emotional expression and stress management, arts engagement fosters emotional regulation and coping (Coholic, 2011; Fancourt et al., 2020). It also enhances a sense of self-awareness and autonomy that contributes to overall well-being (Kim et al., 2023; Jean-Berluche, 2024). Specifically, the arts foster creativity and problem-solving by inspiring others to explore alternatives and solutions, consider diverse perspectives, and build confidence to overcome obstacles (Fancourt & Ali, 2019; Hardiman, 2019). In the social realm, arts-based projects and activities strengthen mutual understanding and appreciation, enhance interpersonal relationships, and promote collaboration among individuals with shared interests (Shukla et al., 2022; Jean-Berluche, 2024). Further, these activities help strengthen social support networks, enhance social connectedness, and foster a greater sense of belonging (Perkins et al., 2021).

Artistic work can enhance violence prevention through the expression of complex emotions and offer insights and education that formal education may not provide. Arts engagement has been found to be a positive channel for adolescents during the pivotal stage of their development (Malin, 2015). Adolescence can be characterized by increased risk-taking behaviors, such as substance use, unsafe sexual activity, school misconduct, and delinquency (National Academies of Sciences, Engineering, and Medicine, 2019; Simon et al., 2022). It is also a critical period for identity exploration, which plays a significant role in shaping long-term academic and career decisions (Branje et al., 2021). Arts-based programs can be both preventive and rehabilitative for youths with behavioral health issues (Office of Juvenile Justice and Delinquency Prevention, 2021). Such approaches may also be particularly beneficial for children and youths involved in child protective services and the mental health system (Coholic, 2011). There is evidence that the arts play an instrumental role in preventing and reducing youth violence, which can potentially decrease antisocial or criminal behaviors (Bone et al., 2022). From a developmental standpoint, arts interventions for youths foster positive development by strengthening self-esteem, self-control, and resilience (Shelton, 2009). Empirically, expressive arts are linked to positive academic, social, and behavioral outcomes for youths (Forrest-Bank et al., 2016), while arts education stimulates curiosity, broadens interests, supports self-expression, and promotes personal growth (Boyes & Reid, 2005).

### 2.2. Using an Arts-Based Approach to Nurture Kindness

An arts-based approach can be used as a universal channel to build empathy, increase understanding, strengthen social bonds, and promote kindness through creative expression (Morizio et al., 2022). Kindness, characterized by acts of compassion and goodwill, is a fundamental value recognized globally (Paat & Lin, 2024). There is evidence that kindness promotion helps improve well-being and quality of life. In the health realm, research evidence suggests that kindness can exert many psychological and physiological benefits, including reducing stress, improving mood, and boosting the immune system (Fryburg, 2022). In particular, engaging in acts of kindness stimulates the release of neurotransmitters such as dopamine and serotonin, along with the hormone oxytocin, all of which contribute to improved mood and enhanced social bonding (Mathers, 2016; Fryburg, 2022; Kucerova et al., 2023). Implementing kindness interventions in healthcare organizations, for instance, has been shown to prevent stress and burnout, foster supportive environments, and construct a culture of respect and benevolence (Greco et al., 2025). Positive psychology interventions that promote psychosocial strengths, such as kindness, have been associated with better pain regulation, improved pain-related outcomes, and enhanced emotional functioning (e.g., more positive affect and less negative affect) (Müller et al., 2016; Braunwalder et al., 2022). Further, when integrated into the form of meditation, loving-kindness has been linked to reduced symptoms of negative emotions (e.g., anxiety and depression) and improved self-reported health (Hofmann et al., 2011; Petrovic et al., 2024). At the community level, promoting kindness helps foster an inclusive society, strengthen communities, and create a welcoming environment for all (Wei, 2023).

### 2.3. Applying an Arts-Based Approach to Foster Civic Learning

Arts-based approaches can also be used to promote civic learning. Civic engagement is essential for strengthening democratic participation, empowering citizens to shape their communities, and addressing local challenges to enhance the quality of life (Powers & Webster, 2021). Aside from its societal advantages, civic engagement has been shown to improve mental and physical health by fostering a sense of purpose, social belonging, and overall life satisfaction (Sagiv et al., 2022). Youth civic engagement strengthens emotional regulation and personal empowerment while promoting active civic and political participation (Martini et al., 2023). Civic education effectively fosters youth participation in civic matters by enhancing their social responsibility and sense of agency, as well as equipping them with skills to engage constructively in their communities (Blevins et al., 2020). These findings highlight the need to integrate civic learning into education and community activities to build healthier, more united, resilient communities. Arts-based civic learning may allow individuals to express their voice, reflect on societal challenges, and collaboratively develop solutions.

### 2.4. Harnessing the Power of the Arts to Encourage Community Egagement

Community engagement plays a significant role in building trust, resilience, and social connections (Lansing et al., 2023). Artistic abilities and talents can serve as a powerful tool for conveying messages and information to the community in order to foster awareness and promote engagement (Fancourt & Finn, 2019). Art exhibitions, for example, can offer valuable insights into a community and serve as a platform for their voices to be heard. Expressive arts can broaden cultural knowledge, celebrate diversity, and foster a deeper appreciation for different perspectives, identities, and lived experiences (Goicoechea et al., 2014). Collaborative creative activities foster meaningful connections and a shared sense of purpose through collective experiences (Jean-Berluche, 2024). In resource-deprived communities, young people often encounter barriers to accessing opportunities for civic engagement (Hoang, 2013). Thus, community-engaged initiatives play a vital role in bridging this gap, allowing young people to connect to their communities and become socially and civically engaged.

## 3. Model Framework

As part of the efforts to help raise awareness and prevent targeted violence along the U.S.-Mexico border and beyond, we integrated an arts-based approach as our project framework for fostering civic learning, critical media literacy, and community engagement. The arts-based approach involves using community-based arts (e.g., visual arts, literary arts, and culinary arts) to foster learning and develop essential connections, mentorship, and/or peer exchange networks to bridge barriers and support dialogues emphasizing social and cultural significance (Ball et al., 2021). The arts-based approach can be used to improve critical media literacy by encouraging the critical examination and analysis of media, promoting transformative social awareness, inquiry, critical thinking, and exploring power differentials in strengthening emergency preparedness (see UCLA Library, n.d.). This approach can also be used to enhance civic learning, including developing civic knowledge, skills, and commitment to interact with the broader society and the civic learning ecosystem (family, peers, schools, the Internet, culture, and institutions). It promotes global awareness and inspires collective action that fosters diversity and inclusiveness (Red & Blue Works, 2019).

## 4. Method

### 4.1. Project Description

*REACH* (Resilience, Education, Action, Commitment, and Humanity) is a community-based project developed in El Paso County, Texas in 2021 that aims to prevent targeted violence. Our project has worked diligently and closely with local experts, health providers, community agencies, shelters, and schools to deliver a broad range of activities and services, including but not limited to our symposium series; training for professionals, paraprofessionals, and community members; classroom presentations; youth workshops; community conversations; civic engagement activities; local health fairs; and a media campaign (Paat et al., 2023). Since October 2023, *REACH* has concentrated primarily on critical media literacy, civic learning, and arts-based approaches to promote awareness and healing across the primary, secondary, and tertiary levels of violence prevention. Located along the U.S.-Mexico border, at the intersection of three states (Texas and New Mexico on the U.S. side and Chihuahua in the Northwestern part of Mexico), El Paso County is home to over 800,000 residents. The U.S.-Mexico border region is characterized by rapid population growth; close familial, cultural, and business connections with Mexico; fluid cross-border mobility; and stark socioeconomic discrepancies. As of the writing of this article (1 May 2025), *REACH* has served over 15,000 participants directly and reached many more individuals via our media campaign (primarily through TV, radio, advertisements inside public transportation, and social media) (Paat et al., 2023). From October 2023 to the present, *REACH* has organized nearly 80 arts-based events/activities of various sizes, engaging over 3000 participants. In addition, it has also launched a podcast series, developed an educational toolkit, organized a series of symposiums and call-to-action summits, and executed a social media campaign.

Using an arts-based approach, our project built trusted partnerships with our project collaborators and the local community to promote communication and meaningful engagement through creative classes and diverse art projects utilizing various mediums and modalities, including painting, drawing, visual sociology, coloring contests, essay writing competitions, graphic design, and culinary arts. Selected end products were showcased at art exhibitions, galleries, and social media outlets to promote a community culture emphasizing social diversity, multiculturalism, and social integration. We also engaged audiences through school events and community classes to expand our reach to a broader audience. Additionally, we offered college students arts-based learning opportunities that strengthen connections to their social surroundings and utilize innovative methods to spread anti-violence messages and promote violence prevention. These activities aim to support at-risk individuals, encourage positive behavioral change, improve mental health, and build resilience.

### 4.2. Data Collection

This study employed a descriptive qualitative design with an evaluative focus to offer a comprehensive and detailed account of the community partners’ experiences and perspectives, while examining the effectiveness and impact of our project. In this study, we interviewed 15 community partners (from schools, shelters, and a juvenile rehabilitation program) who collaborated with our project in organizing events and activities using an arts-based approach to promote kindness awareness and violence prevention since the inception of the project. Interview data were collected between May 2024 and October 2024. Once informed consent was obtained, the community partners were asked questions related to their experience in our collaboration and strategies for fostering a culture of kindness, reducing incidents of targeted violence, and encouraging a greater sense of civic duty. Because some of our community partners had either left their original roles or been reassigned to different units, our pool of interviewees was limited. However, we were still able to interview the majority of our community partners in order to ensure a broad and diverse perspective for this study. The interviews took about 20 min on average. Each interviewee was offered a USD 15 gift card as a token of appreciation for their time and contribution. The average age of the interviewees was 46.6 years old. All interviewees were female and had a college education. About 60 percent of them had at least one master’s degree. With the exception of one who is African American, the others self-identified as Hispanic, Latina, Mexican, or Mexican American. To protect the identities of our interviewees, each was assigned a participant identification number instead of using their real name. To prevent confusion, we used the term “interviewees” to refer to the community partners we interviewed in this study and “participants” to indicate the audiences engaged in the arts-based activities/events in this article.

### 4.3. Data Analysis

Interview recordings were transcribed verbatim before they were analyzed using NVivo 15^®^. The data were analyzed using thematic data analysis, a qualitative research method to analyze and interpret patterns within textual data by systematically coding the data and grouping the codes into themes to derive deeper meanings and insights (Naeem et al., 2023). This approach is prevalently used in the field of social sciences to understand subjective experiences or perspectives. We conducted the data analyses both inductively, by allowing the themes to emerge, and deductively, where our interview questions and predefined themes guided the analyses (Dawadi, 2020). Transcripts were analyzed line by line, and three research team members independently analyzed the data. Relevant data segments were systematically coded and assigned labels before they were grouped into broader themes based on similarities and recurring patterns (Maguire & Delahunt, 2017). In cases of discrepancies, the research team met to discuss the inconsistencies and resolve differences.

## 5. Findings

In sum, we grouped our community partners’ responses into the following four themes: (1) the inherent benefits of the arts, (2) promoting kindness and preventing violence through artistic expression, (3) teaching civic responsibility through the arts, and (4) practical strategies for collaborating with community partners.

### 5.1. The Inherent Benefits of the Arts

Consistent with the existing literature attesting to the mental health benefits of arts engagement (Van Lith et al., 2012; Jensen & Bonde, 2018), community partners emphasized several mental health benefits of arts engagement, such as stress reduction, mood enhancement, increased self-compassion, and improved emotional well-being. Additionally, arts engagement improved physical health, particularly for older participants, by enhancing their fine motor skills and hand-eye coordination. The quotes below illustrate some of the community partners’ perceptions:

I think what they get out of it is a little bit of self-love. I feel like they … feel a little more content, … a little bit more joyous and comfortable within themselves … the comfortability of coming together and building something and being able to share an experience or being able to share laughter.

(P3)

Sometimes, if they’re feeling … depressed or sad, … you (painting wooden crafts that convey positive messages) bring some hope or some sunshine.

(P11)

Arts engagement also served a therapeutic purpose (Beerse et al., 2020), where participating in artistic activities contributed to enhanced mindfulness, allowing the participants to focus on the positive aspects of life rather than dwelling on the negative, as depicted in the following quotes:

Working on painting is therapeutic for them and also the classes … creating peace and recognizing what is violence [*sic*].

(P2)

Most of them (homeless shelter residents) … they express their emotions in different ways. … Having these … activities … it takes out all stress … try not to think about anything other than what they are doing and just focus on this (the present).

(P6)

You’re creating … awareness through art. … It’s kinesthetic. … You are touching. You are feeling. And you are using your senses. … It’s a full implementation of other senses.

(P7)

The arts were both enjoyable and stimulating activities that nurtured creativity. For students, engaging in artistic projects fostered a sense of pride in their work and filled them with excitement as they showcased their creations to their teachers and parents. The quotes below highlight the interviewees’ observations, sharing the fun they witnessed from the participants:

They had the opportunity to show that creativity while sending out a positive message. … That art activity … was amazing for them.

(P4)

I think [it] really resonated with the kiddos [*sic*]. … They had so much fun and they were excited about being in the gallery and all that. So, it was really nice.

(P5)

They want to have that feeling of, “Uh, I’m able to give something to someone.” “Someone’s going to be able to see what I did.” And they take pride in it.

(P14)

Drawing and painting gave participants a unique and creative way to express their diverse perspectives on kindness within their community. This was exemplified by one of the school staff members, who highlighted the students’ various ways of interpreting, sharing, and spreading kindness through the arts.

It was insightful to see what’s in their little brains, like how they identify kindness. … Some of them identify it through friendship. Some of them through family, through animals, through the world, so … you can kind of get an inside [*sic*] on how their little brains are working and what they think is valuable.

(P5)

In resource-limited communities, the arts could serve as a valuable means of enrichment by providing opportunities for personal growth and cultural engagement that might otherwise be inaccessible, as highlighted by a shelter staff member:

The children … the only involvement they ever get is the ones that are in school. So when they come and they do projects, activities, events here in the shelter, it’s very rewarding for them. And it gets them to be themselves. … They’re enjoying themselves for who they are.

(P10)

Arts engagement fostered social connections by reducing isolation and linking participants to the real world (Perkins et al., 2021). Collaborative creative activities offer support, camaraderie, and a strong sense of community (Jean-Berluche, 2024). Additionally, engaging in arts activities provided an opportunity to relax and temporarily escape from their hardships. Engaging in arts activities, such as cupcake decorating and painting, fostered a sense of community and a team-building experience within the shelters. Some of our community partners from the shelters disclosed that their residents often keep to themselves despite living under the same roof but “came out of their shells” and became more socially engaged and more eagerly participated in the activities. The following quotes exemplify this.

They were also doing it (art activities) with their moms, … so I think that was a really good bonding experience for everybody.

(P9)

I’ve seen … some of the women that [*sic*] don’t necessarily come out; a lot of them have already come out and started … talking more, interacting more with the women, so it does help.

(P12)

I can talk about the cake decorating. … All the ladies were able to decorate … cupcakes and kind of talked to each other … co-existed, talked about how they feel, and … how things are going … and kind of … bring [*sic*] together the women. And it was really nice.

(P3)

### 5.2. Promoting Kindness and Preventing Violence Through Artistic Expression

The arts can be an impactful tool for inspiring acts of kindness, cultivating empathy, and sharing uplifting messages (Morizio et al., 2022). We used the arts to simultaneously educate participants on civic values (e.g., kindness, empathy, respect, responsibility, honesty, and cooperation) and help individuals gain the cognitive, emotional, and social benefits of artistic engagement. Through our arts-based projects, participants could use their painting to highlight the importance of caring for each other, bringing diverse groups of people together, and fostering unity. The arts could also be used for emotional reflection, allowing participants to process their reflections in a supportive and non-judgmental way and build a deeper sense of self-awareness. Through painting, coloring, and writing, the arts helped cultivate mutual respect and understanding and contributed to a more thoughtful and caring community. During the activities, participants were able to reflect on the concepts of kindness and peace via their creative works. The activities also allowed them to visualize and interpret what these concepts mean to them personally and collectively and encouraged them to practice, advocate, and integrate these concepts into their day-to-day lives. In response to our project’s goal to promote kindness and prevent violence, we sought input from community partners to gain their insights on effective strategies for promoting kindness and engagement. Below are quotes that align with and enhance the arts-based approach and complement its core principles.

Continuing to have more activities, maybe guest speakers. … When it’s someone else talking to the kids, that will motivate the kids and engage them. They listen cause [*sic*] it’s someone outside of the school.

(P4)

If kids hear it in some different ways, … it fosters that kindness in the school. They’re not just hearing it from us, they’re hearing it from others, … agencies outside our school. So, it makes sense to them that kindness is something that … should be implemented not only at school but at home, everywhere else.

(P7)

One way to foster compassion is by preventing violence both in person and online. When discussing ways to promote kindness, education emerged as a key priority to help younger children develop critical thinking skills. Our project prepared information packages for all child participants as we have been told that providing children with a tangible takeaway can serve as a reminder, especially given the abundance of information they receive from our facilitators and guest speakers. Below are several quotes that can be thoughtfully integrated into arts-based activities to enhance the overall experience.

Teaching children to respect one another from the beginning and … staying away from hatred. … Children are like sponges and they absorb everything … and they internalize that. So, teaching them “No! … respect is better, working together is better.”

(P8)

Making kids aware of what’s wrong, what’s … right. … And … one of our core values is accountability, students need to be made aware that … they must be accountable for their actions and violence should never be … the resort. So, making kids aware of different choices.

(P7)

Sometimes kids do things and … they’re not fully aware of what it is. Like bullying, for example. So, they might think, “Oh, that? We’re just teasing each other,” but it could be seen as bullying. So, I think … educating them so that they know exactly what it is, and then providing tools and resources so that it doesn’t happen.

(P15)

**Figure 1:**
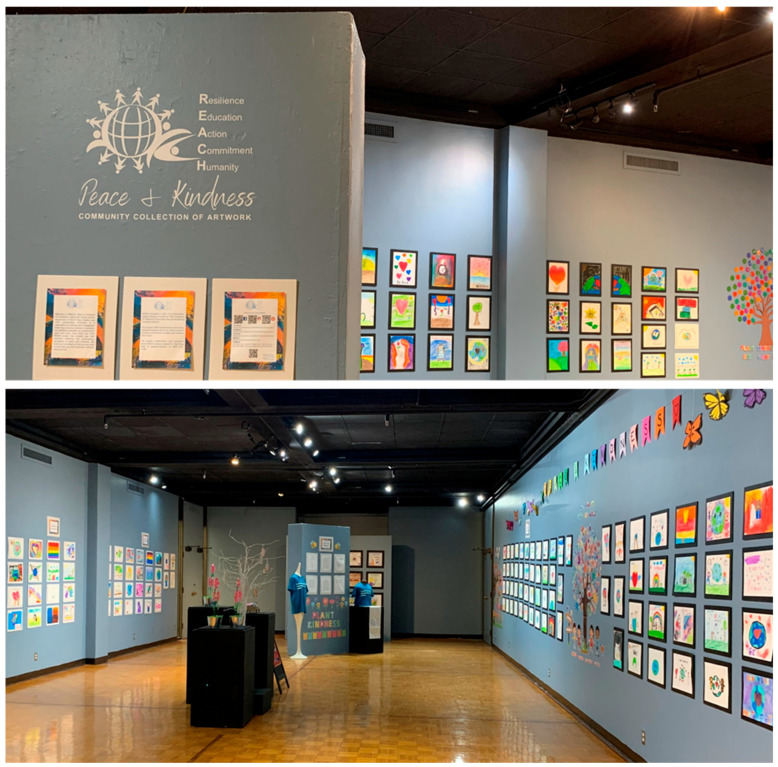
REACH’s Peace & Kindness Art Exhibition 2024.

**Figure 2:**
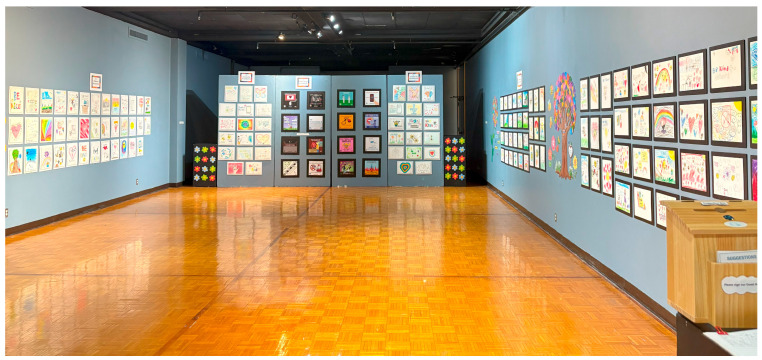
REACH’s Peace & Kindness II Art Exhibition 2025.

Additionally, hosting community events provided a valuable opportunity to raise awareness, engage the community, increase exposure to important issues, and create a sense of collective responsibility to promote social change. Our two art exhibitions (Figure 1 and Figure 2) attracted hundreds of visitors over the course of two weeks and one week, respectively. One of the community partners shared her perspective:

Having … events that promote kindness and … spread love, and kind of … set some sort of motion within the community. … I think events that … give out positivity … would be really cool.

(P3)

### 5.3. Teaching Civic Responsibility Through the Arts

Arts activities can serve as powerful tools for engaging communities while fostering civic responsibility and raising social awareness (Chung et al., 2009; Lee et al., 2020). Through arts engagement, communities can engage in meaningful conversations and encourage active participation in social causes. Artistic expression may be integrated into civic education to inspire the newer generations to contribute positively to their communities. In our project, we utilized arts-based activities to teach school-age children about civic responsibility while highlighting themes of kindness, compassion, and inclusion. Additionally, we hosted art exhibitions (Figure 1 and Figure 2; REACH Violence Prevention, 2024, 2025) to spark meaningful dialogue and inspire a sense of collective responsibility within the community. To organize our exhibitions, we collected artworks from different schools, shelters, and our project events to reinforce that everyone’s voice matters. Additionally, we created a space where community members could leave messages to inspire collective action in the exhibitions. Further, we leveraged social media content—such as infographics, social media posts, and videos—along with campaigns to effectively spread messages and raise awareness through digital art. Below, our community partners shared the importance of using a community-based approach and modeling in teaching civic responsibilities:

I think our kiddos [*sic*], from what I’ve seen, tend to live in a very narrow world. … I think getting them more involved in the community and out of their phones and technology … would be very helpful.

(P5)

Maybe … an act of kindness … where … they’re put to … make something. Maybe a craft, maybe a little gift, … and they go out and share it with the community. … I think that’s one of the ways … you can show civic kindness.

(P3)

We’re trying to create an environment of intrinsic motivation. … You should always behave a certain way not just because you’re gonna [*sic*] receive a reward but because it’s the right thing to do. … This is our main focus in the school.

(P7)

### 5.4. Practical Strategies for Collaborating with Community Partners

We learned that working collaboratively with community partners helps create a meaningful and sustainable impact. As part of our evaluation efforts to enhance the project, we invited our community partners to share their insights and experiences about our collaboration. We grouped their responses into three main sub-themes—organization/structure, quality of engagement, and challenges. The community partners were generally very grateful that our project was “thoughtful about what we can do,” along with its strong coordination and communication, as illustrated by one of the community partners:

I know that you guys … were very deliberate … with what kind of lessons you brought in, the materials, the resources you provided. Everything was very well organized. I think that *REACH* did an awesome job at keeping track of the numbers coming in and … setting up and the way … we were able to reach every single student I think was awesome!

(P1)

The community partners also appreciated the project’s flexibility, as the project team was willing to accommodate their schedules and the needs of the organizations and schools and made themselves readily available. Punctuality was key to ensuring things were well planned and speakers were on time. Sending gentle reminders and reliably following up also helped ensure that tasks stayed on track and deadlines were met. Additionally, consistency was another key factor our community partners found critical:

Consistency is very important to them (shelter residents). … Even though you have one person here, you are here, so … they know that they can count on you. … And I believe that has a lot to do with … being … truthful to what you do.

(P11)

Other key qualities include patience, a mission-driven mindset, responsiveness, organization, and attention to detail. One of the community partners shared her perception:

You all as a…[team] are very organized and detailed, and … very efficient. And … you can tell you love what you do. So … it has been motivating but also really nice to work with you all.

(P5)

The treatment of the community partners is equally important. Having team members who were approachable, supportive, and genuinely interested fostered positive relationships, as illustrated by one of the quotes:

[Project team member] … does her homework. She follows up. She asks a lot of really great questions because she is coming in from the outside, so she’s very prepared. She is always asking questions about what can be done, what can’t be done, … what my thoughts are. … So, it has been a good experience.

(P14)

The success of an arts-based project also relies on its alignment with the organization’s core values and mission. It is critical that the organizations and arts-based project share common values and mission. Key values considered vital to our community partners included kindness, empathy, integrity, responsibility, and caring for others, as depicted in the quotes below:

I did … like the fact that *REACH* … focuses a lot on the character values. … We are actually … a national character school. So … the fact that … the agency outside of school was able to help us bring some … workshops for the kids, it worked very well. … It was … good integration of resources.

(P7)

For me as a school counselor, I promote … the empathy portion. I promote for kids … to be kind to one another … using their words. … It (the activity) just goes hand in hand with what I’m doing, our mission … to provide that safety net for our students. … And I think it just matched our goals.

(P1)

So everything that you guys … are doing and teaching kind of falls in line with our school’s mission and vision. … Your kindness, lessons, and activities fit right in to what we’re already trying to teach them here.

(P15)

As with any community-based project, collaborations and partnerships inevitably face challenges. We also asked our community partners to discuss the challenges that they have encountered throughout our collaboration. Scheduling conflicts tended to be one of the most commonly raised challenges, as illustrated below:

I’d say … arranging the time, and … that’s not necessarily on your end but more on the campus end. … Finding the time … the dates, the time the classes that we were working with … so it doesn’t impede too much with instruction, but that’s more on the campus end.

(P4)

As a school, I know … our schedules are crazy and there are last-minute changes, and even with that, you guys have been very patient. And you’ve been able to work with us … because things just come up or administration will make changes at the last minute. So, I’m grateful for that as well.

(P5)

Sometimes, even if community partners believed that the activities our project brings benefit the community they serve, they still needed to convince their client population and secure buy-in from the administration, as revealed by one of the shelter staff members:

It’s hard for the mothers and the children to envision the benefits of things that they never experience. … Trying to convince them, trying to show them this is great—it’s not a chore, it’s not boring. … It’s something that is gonna [*sic*] help you. That, you know, is kind of tricky but you guys have been able to do that.

(P8)

## 6. Discussion and Conclusions

Empirical evidence suggests that the arts can have transformative benefits for both individuals and communities (Forrest-Bank et al., 2016; Perkins et al., 2021; Jean-Berluche, 2024). Drawing on these benefits, we integrated an arts-based approach to actively engage the community in civic learning, media literacy acquisition, and violence prevention. This project can serve as a foundational framework for launching and implementing community-based projects and initiatives along the U.S.-Mexico border and beyond. Leveraging an arts-based approach to engage the community in promoting positive causes and preventing violence can offer substantial benefits. The advantages gained at the community level may have a ripple effect, creating broader influence by reaching individuals and various segments within the community. Overall, our community engagement efforts using an arts-based approach have created numerous opportunities and rewarding experiences for local communities with limited access to the arts. However, implementing these activities was not without its challenges and obstacles. In what follows, we offer constructive insights and lessons learned that may prove valuable for others undertaking similar initiatives in the future.

### 6.1. The Power of Creative Expression

The arts are a universal medium that empowers individuals to share their emotions, experiences, and viewpoints in ways that connect and inspire. The arts provide a means to help with self-expression and the communication of ideas and feelings (Lindsey et al., 2018). They may also be used to increase communication, problem-solving skills, and efficacy (Fancourt & Ali, 2019; Hardiman, 2019; Jean-Berluche, 2024). Our study highlights that engaging in creative arts activities can elevate mood, alleviate stress, and foster a strong sense of connection. Indeed, empirical evidence postulates that expressive arts can be used for self-discovery and change as an intervention in art therapy (Vaartio-Rajalin et al., 2021). From the therapeutic perspective, the arts offer a creative means to support survivors’ psychological well-being, helping them process their experiences and come to terms with overcoming adversity (Hass-Cohen et al., 2018). Coholic (2011) highlighted the use of arts-based methods to teach mindfulness-based methods to young people. In addition to enhancing students’ motivation, emotional well-being, and achievement (Guo, 2024), there is evidence that creative art activities also provide incarcerated youths with a platform for positive change (Ezell & Levy, 2003; Office of Juvenile Justice and Delinquency Prevention, 2021) and serve as part of a strategic, community-level effort to prevent dropout (Charmaraman & Hall, 2011).

### 6.2. Strengthening Accessibility and Inclusion

Our study found that the arts are a valuable tool for strengthening community bonds. Collaborative arts projects can encourage dialogue that increases mutual understanding. In our project, we used the arts as a platform to enhance civic awareness, educate participants on civic responsibility, and inspire greater civic engagement. To promote inclusivity and representation, our project helped provide marginalized and underrepresented communities opportunities to engage in enrichment activities that otherwise were not available. Our arts-based activities utilized an inclusive approach that ensured people, regardless of age, background, or race, could participate, making it an ideal approach for community engagement. This approach also allowed our project to adapt to community needs and adjust based on our community partners’ input and ensured that our project was responsive to the local community’s evolving needs and challenges. We recommend that community projects consider cultural relevance by integrating local traditions and artistic practices into their programming. Future studies may explore recreational activities tailored to the needs of racially and ethnically diverse communities with limited resources. We suggest that policymakers expand youths’ access to creative arts activities in economically disadvantaged communities, foster parental involvement, and encourage participation in open house events.

### 6.3. Supporting Sustainability and Longevity

Our project acknowledges the importance of supporting the sustainability and longevity of community-based projects to ensure that arts-based projects can have consistent and long-term impacts. We also note the importance of staying consistent with the project to allow time for the project to evolve and adapt. While we recognize that artistic products are valuable, we must also value the engagement process equally. Specifically, promoting meaningful dialogue, shared ownership, and inclusive participation can have long-lasting impacts (Allen et al., 2024). It is crucial to recognize that arts-based programming must be consistent and long-term to effectively support participants (Berdychevsky et al., 2019). For community engagement to create a genuine and lasting impact, it must extend beyond short-term projects, prioritizing capacity building and long-term sustainability. Temporary programs that come and go create disruptions, as communities require reliable, ongoing programming to thrive. Research on stakeholder engagement highlights that community partnerships are most effective when they prioritize authentic representation, open communication, and collaboration from the beginning of the partnership (Allen et al., 2024). This involves ensuring that community members have a meaningful voice in decision-making, rather than merely being recipients of intervention. We recognize the challenges of balancing the goal of long-term sustainability with the current reality that many of our events are single occurrences. While we have limited engagement with participants, our project focuses on cultivating deeper connections with community partners with whom we have established relationships.

## 7. Limitations

Thus far, we have explored the benefits of incorporating the arts to foster kindness, prevent violence, promote civic responsibilities, and enhance community engagement. However, it is essential to acknowledge certain limitations of this study. Since this study relied on the perspectives of 15 female community partners and did not include participant tracking, measuring the long-term therapeutic effects of the arts is beyond the scope of this paper, particularly since most *REACH* events are one-time engagements with some events involving more than a hundred participants. Furthermore, the primary goals of this project are to expand access to the arts and foster community engagement rather than to assess individual benefits. Given that we interviewed experienced community partners who had direct involvement and regular contact with participants, we anticipate their insights into effective community strategies will be invaluable. Like many other studies of primary data collection, our data were subjected to self-selection factors where community partners who had a cordial collaborative relationship with our project might be more likely to participate in the interviews. Since our project can only support a limited number of organizations and schools, given the constraints of our resources (i.e., funding, labor, and time), the number of community partners available for interview (i.e., sample size) is limited. We encourage that future research broadens the diversity of their interviewees by considering a wider range of demographics (e.g., genders, race, and ethnicity), and experiences beyond the shelters, schools, and the criminal justice system. We noted that the aim of this study is not to produce research-based conclusions but to offer a translational framework that provides insightful directions for others in their arts-based community engagement efforts, particularly in the areas of violence prevention and other meaningful causes. We hope that our community-based initiative inspires others to adopt similar approaches to support meaningful causes in their communities.

## Data Availability

Data are not available for public dissemination to protect the privacy of the interviewees.
